# Icariin Improves the Viability and Function of Cryopreserved Human Nucleus Pulposus-Derived Mesenchymal Stem Cells

**DOI:** 10.1155/2018/3459612

**Published:** 2018-06-27

**Authors:** Sheng Chen, Xiangyu Deng, Kaige Ma, Lei Zhao, Donghua Huang, Zhiliang Li, Zengwu Shao

**Affiliations:** Department of Orthopaedics, Union Hospital, Tongji Medical College, Huazhong University of Science and Technology, Wuhan 430022, China

## Abstract

Nucleus pulposus-derived mesenchymal stem cells (NPMSCs) have shown a good prospect in the regeneration of intervertebral disc (IVD) tissues. However, fresh NPMSCs are not always readily available for basic research and clinical applications. Therefore, there is a need for an effective long-term cryopreservation method for NPMSCs. The aim of this study was to determine whether adding icariin (ICA) to the conventional cryoprotectant containing dimethyl sulfoxide (DMSO) had a better cryoprotective effect for NPMSCs. The results showed that the freezing solution containing ICA along with DMSO significantly increased the postthawed cell viability, decreased the apoptosis rate, improved cell adherence, and maintained the mitochondrial functions, as compared to the freezing solution containing DMSO alone. And the inhibition of oxidative stress and upregulation of heat shock proteins (HSPs) in the presence of ICA also confirmed the beneficial effect of ICA. Furthermore, ICA had no cytotoxicity and did not alter the characteristics of postthawed NPMSCs. In conclusion, these results suggested that the addition of ICA to the conventional freezing medium could improve the viability and function of the cryopreserved human NPMSCs and provided an optimal formulated freezing solution for human NPMSC cryopreservation.

## 1. Introduction

Low back pain (LBP) is one of the most common health problems throughout the world, which creates heavy financial burden globally [1]. Intervertebral disc (IVD) degeneration is considered as the main cause of LBP [2]. However, surgical operations and conservative treatments for IVD degeneration are not long-lasting and effective for the limitation that they cannot restore the IVD tissues [3]. In the past decade, supplementation with exogenous mesenchymal stem cells (MSCs), such as MSCs derived from bone marrow and adipose tissue, has shown positive outcomes for the repair of IVD degeneration [4]. And in recent years, evidence has been found that endogenous nucleus pulposus-derived mesenchymal stem cells (NPMSCs) exist naturally in the IVDs [5–7]. As a novel approach to repairing disc degeneration, application of endogenous NPMSCs has shown exciting prospect and attracted increasing attention [8, 9]. However, freshly harvested and viable NPMSCs are not always available for regenerative medicine. Thus, the effective and long-term preservation of NPMSCs is crucial for the application of NPMSCs.

The cryopreservation is the most important and widely used technology for long-term preservation of MSCs. Currently, the existing cryopreservation methods for MSCs can be classified into two main categories, slow freezing and vitrification (rapid freezing) [10]. But both methods have some notable limitations [11], and there is an increasing amount of evidence showing the adverse effects of conventional cryopreservation methods for MSCs, such as the cytotoxicity of the standard cryoprotectant dimethyl sulfoxide (DMSO), cell apoptosis, cellular structure damages, and oxidative stress [12–14]. Recently, many approaches have been proposed for a more efficient application of cryopreserved MSCs, among which addition of selected agents in cryopreservation solution has shown good application prospects [15, 16].

**Figure 1:**
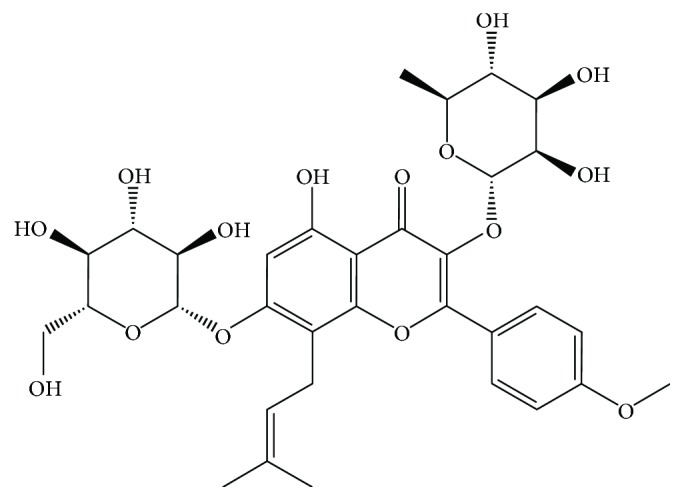
The chemical structure of ICA.

Icariin (ICA) is a main active ingredient extracted from the stem leaf of *Epimedium brevicornum* Maxim [17], and its chemical structure is shown in Figure 1 [18, 19]. A large number of in vitro and in vivo studies suggested that ICA could scavenge reactive oxygen species (ROS) [20] and exert antioxidative function in protecting the brain and heart [21–23]. Furthermore, ICA demonstrated other extensive pharmacological effects such as antiapoptosis effect, reproductive effect, anti-inflammation effect, and immunoprotective effect [24, 25]. Due to its extensive cell protective effects, we hypothesized that ICA could be used in cryopreservation of NPMSCs. Therefore, the present study aimed to evaluate the effect of ICA on cryopreserved human NPMSCs.

## 2. Materials and Methods

### 2.1. Reagents and Antibodies

ICA (>98% purity) was purchased from Nanjing Zelang Pharmaceutical Technology (Nanjing, China). DMSO was purchased from Sigma. The standard MSC expansion medium, osteogenesis kit, adipogenesis kit, and chondrogenic kit were purchased from Cyagen Biosciences Inc. (Guangzhou, China). Cell counting kit-8 (CCK-8) was purchased from Dojindo (Japan). TUNEL staining was purchased from Roche Diagnostics GmbH (Roche, Germany). Phalloidin conjugated with Alexa Fluor 488 and DAPI was purchased from Sigma. Annexin V-FITC/PI apoptosis detection kit, lactate dehydrogenase- (LDH-) cytotoxicity assay kit, JC-1 staining, ROS detection kit, glutathione peroxidase (GPx) assay kit, superoxide dismutase (SOD) assay kit, lipid peroxidation malondialdehyde (MDA) assay kit, and western and IP cell lysis kit were purchased from Beyotime Institute of Biotechnology (Beyotime, China). Primary antibodies against heat shock protein (HSP) 90, HSP70, and HSP27 were purchased from Santa Cruz Biotechnology Inc. Primary antibody against *β*-actin and all secondary antibodies used for western blotting were purchased from Proteintech (Wuhan, China). Fluorescently tagged human monoclonal antibodies were purchased from BD Pharmingen (Becton Dickenson, San Jose, CA).

### 2.2. Isolation and Culture of NPMSCs

The human nucleus pulposus (NP) samples were obtained from five patients who had undergone discectomy for degenerative disc disease, and the details of all patients are shown in Supplementary Table S1. All procedures were approved by the Clinical Research Ethics Committee of Tongji Medical College, Huazhong University of Science and Technology. The NPMSCs were isolated and cultured as described previously with minor modifications [26, 27]. After being washed with phosphate-buffered saline (PBS), NP tissues were dissected and digested with 0.2% type II collagenase at 37°C overnight. Then, the partially digested tissue and liberated cells were collected by centrifugation at 300*g* for 5 min and cultured in the standard MSC expansion medium, consisting of Dulbecco's modified Eagle's medium-low glucose (DMEM-LG), 10% fetal bovine serum (FBS), 2 mmol/L l-glutamine, and 1% penicillin/streptomycin at 37°C with 5% CO_2_. After 24 hours, fragments and suspended cells were removed, and the adherent cells were fed by complete replacement of medium every other day. The cultures were 1 : 2 subcultured when cells reached confluence of 80–90%. Cells at the third generation were used in this study. And the details about the use of samples are shown in Supplementary Table S2.

### 2.3. Cryopreservation and Thawing

The cryopreservation process was performed as described previously [28, 29]. NPMSCs were trypsinized, washed, and resuspended at 10^6^ cells/mL in different freezing medium formulations containing 10% DMSO or combination of 10% DMSO and 25 *μ*M ICA as a cryoprotectant in DMEM-LG supplemented with 20% FBS. The cell suspension (1 mL) was transferred to each cryovial and then placed in a controlled-rate freezing container. When achieving −80°C, the cryovials were removed from the container and stored in liquid nitrogen. One week later, the NPMSCs were taken out of the liquid nitrogen and thawed quickly in a water bath at 37°C and then transferred into the standard MSC expansion medium for centrifugation at 300*g* for 5 min. The pelleted NPMSCs were resuspended and cultured in the standard MSC expansion medium, and about 80% cells were obtained after thawing. The groups used in the experiment were as follows: (I) the noncryopreserved NPMSCs were used as control, (II) DMSO, (III) DMSO + ICA.

### 2.4. CCK-8 Assay

NPMSCs were seeded in a 96-well plate (0.5 × 10^4^ per well) and then treated with different concentrations (0, 6.25, 12.5, 25, 50, and 100 *μ*M) of ICA for indicated times (1, 3, 5, and 7 days). And the cell proliferation was determined by CCK-8 assay to evaluate the cytotoxicity of ICA. To determine the effect of ICA on the cell viability of the noncryopreserved NPMSCs and postthawed NPMSCs, cells with different treatments were seeded in a 96-well plate and cultured for 24 h. Then, the cell viability was determined by CCK-8 assay. The absorbance detection at 450 nm was used.

### 2.5. Observation of Cell Morphology

After 24 h of thawing, the cells were observed under the inverted microscope (Olympus, Japan). To further determine the cell apoptosis, more sensitive TUNEL staining was used. The NPMSCs were fixed in 4% paraformaldehyde for 1 h and then permeabilized with 0.1% TritonX-100 for 10 min. Next, the cells were washed twice with PBS and incubated with TUNEL staining for 1 h at 37°C in the dark, following the manufacturer's protocol. Apoptotic cells were observed under the inverted fluorescence microscope (Olympus, Japan).

### 2.6. Apoptosis Rate

Cell apoptosis rate was measured by Annexin V-FITC/PI apoptosis detection kit following the protocol as described previously [30]. Briefly, the NPMSCs were resuspended in 500 *μ*L binding buffer after 24 h of thawing, and 5 *μ*L Annexin V-FITC and PI were added. Then, the samples were incubated in the dark for 15 min at room temperature. The apoptosis rate was analyzed by flow cytometry (BD LSR II, Becton Dickinson).

### 2.7. Cell Adherence and Filamentous Actin Distribution

The NPMSC adherence on the coverslip surface was determined by measuring the distribution of cytoskeleton filamentous actin (F-actin) stained with phalloidin conjugated with Alexa Fluor 488. The postthawed NPMSCs were grown over a coverslip for 24 h and fixed in 4% paraformaldehyde. Then, the cells were permeabilized with 0.5% TritonX-100. After being washed twice with PBS, the cells were stained with phalloidin conjugated with Alexa Fluor 488 and DAPI, respectively. The stained cells were imaged with a confocal laser scanning microscopy (LSM, Zeiss, Germany).

### 2.8. LDH-Cytotoxicity Assay

The noncryopreserved MSCs and postthawed MSCs were seeded in a 96-well plate and cultured for 24 h. The culture supernatants were collected, and the release of LDH was analyzed by a LDH-cytotoxicity assay kit, according to the manufacturer's instructions.

### 2.9. Mitochondrial Membrane Potential (MMP) Assay

JC-1 is a cationic dye that exhibits potential-dependent accumulation in mitochondria, indicated by a fluorescence emission shift from green to red. When MMP reduces, the ratio of red/green fluorescence will decrease. A JC-1 staining kit was used to measure the MMP in this study. The MMP assay staining solution was made by mixing the standard MSC expansion medium and JC-1 working solution at 1 : 1 (*v*/*v*). The noncryopreserved MSCs and postthawed MSCs were incubated in the staining solution in the dark at 37°C for half an hour. Then, the cells were resuspended in staining buffer (1x) and measured for MMP by flow cytometry. The values of MMP were expressed as ratio of red over green fluorescence intensities.

### 2.10. ROS Assay

The intracellular ROS level was measured using a ROS detection kit. The noncryopreserved MSCs and postthawed MSCs were incubated with 2,7-dichlorofluorescin diacetate (DCFH-DA) in the dark at 37°C for 30 min. Then, the cells were washed twice with PBS and measured for ROS production by flow cytometry (BD LSR II, Becton Dickinson) following the manufacturer's instructions.

### 2.11. Intracellular Oxidation Product and Reductive Substances Assays

The cells were lysed on ice using a western and IP cell lysis kit. The protein concentration was measured with a BCA protein assay kit. The activities of GPx and SOD and the cellular content of MDA were analyzed according to the manufacturer's instructions.

### 2.12. Western Blot Analysis

The cells were lysed on ice with a western and IP cell lysis kit. Then, the protein extracts were collected by centrifugation at 12,000 ×g for 10 min at 4°C. After protein transfer, the membranes were blocked by nonfat milk and then incubated overnight at 4°C with human polyclonal antibody against HSP90 (1 : 1000), HSP70 (1 : 1000), HSP27 (1 : 1000), and *β*-actin (1 : 5000). After washing for three times, the membranes were incubated with secondary antibodies for 1 h in the dark at room temperature. Finally, the proteins were detected using the enhanced chemiluminescence method following the manufacturer's instructions.

### 2.13. Surface Marker Identification

The surface markers of MSCs were identified by flow cytometry following the protocol as described previously [26]. The collected cells were washed with PBS and stained with fluorescently tagged human monoclonal antibodies against CD105, CD73, CD90, CD34, and HLA-DR. Then, the labeled cells were examined via flow cytometry (BD LSR II, Becton Dickinson).

### 2.14. Multilineage Differentiation

To assess the multilineage differentiation potential of NPMSCs, the osteogenic, adipogenic, and chondrogenic differentiation was induced. For osteogenic differentiation, the NPMSCs were stained with the Alizarin Red after 21 days culture following the protocol of the osteogenesis kit. For adipogenic differentiation, the NPMSCs were stained with the Oil Red O after 14-day culture following the protocol of the adipogenesis kit. For chondrogenic differentiation, the NPMSCs were stained with the Toluidine Blue after 21-day culture following the protocol of the chondrogenic kit.

### 2.15. Statistical Analysis

Statistical analysis was conducted using GraphPad Prism 6 software (GraphPad Software Inc., San Diego, CA). All data were obtained from at least three independent experiments and presented as mean ± standard deviation (SD). Multiple sets of data were analyzed by one-way analysis of variance (ANOVA) test, followed by Tukey's post hoc test. Student's *t*-tests were used in the analysis of two-group parameters. Statistical significance was set at *P* < 0.05.

## 3. Results

### 3.1. ICA Increases the Cell Viability of Postthawed NPMSCs

**Figure 2:**
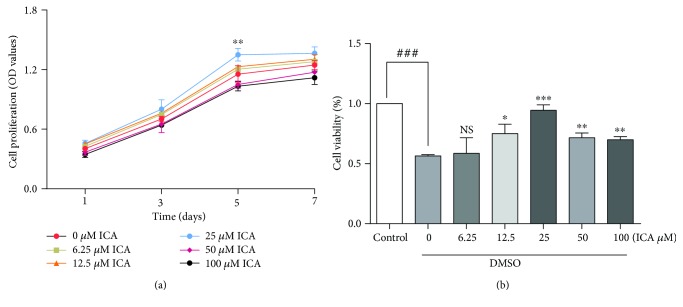
Cell proliferation and cell viability of NPMSC measuring by CCK-8. (a) NPMSCs were treated with different concentrations (0, 6.25, 12.5, 25, 50, 100 *μ*M) of ICA for indicated times (1, 3, 5, 7 days). (b) Different concentrations (0, 6.25, 12.5, 25, 50, 100 *μ*M) of ICA was added to the conventional cryoprotectant to verify the cryoprotective effects of ICA. NS means no statistical significant difference. The data are expressed as mean ± SD from three independent experiments. (^###^ *P* < 0.001 versus control; ^∗^ *P* < 0.05, ^∗∗^ *P* < 0.01, ^∗∗∗^ *P* < 0.001 versus 0 *μ*M of ICA).

As shown in Figure 2(a), ICA showed no cytotoxicity toward NPMSCs. To evaluate the effect of ICA on the cell viability of postthawed NPMSCs, a CCK-8 assay was performed. The results demonstrated that the viability of NPMSCs cryopreserved with DMSO was 56.46 ± 0.66% of the control group (Figure 2(b), *P* < 0.001). For NPMSCs cryopreserved with DMSO and ICA, ICA (12.5–100 *μ*M) significantly increased the viability of postthawed NPMSCs (*P* < 0.05), with maximum viability observed at a concentration of 25 *μ*M. The concentration (25 *μ*M) of ICA was used in the following experiments.

### 3.2. Protective Effect of ICA on Apoptotic Cell Death in Postthawed NPMSCs

**Figure 3:**
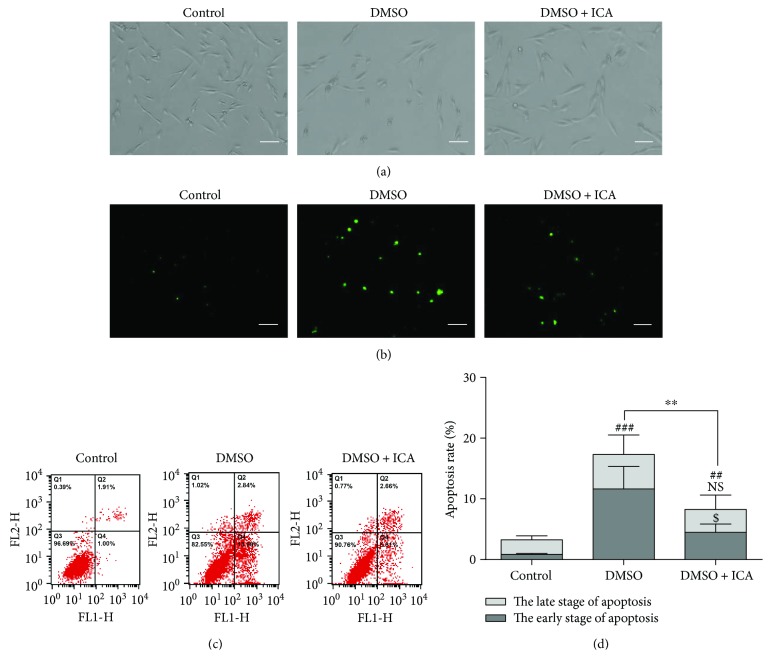
The antiapoptosis effect of ICA on postthawed NPMSCs. (a) The phase-contrast photomicrograph of NPMSCs (bar = 100 *μ*m). (b) TUNEL staining of NPMSCs (bar = 100 *μ*m). (c) Representative images of cell apoptosis by flow cytometry analysis after Annexin V/PI dual staining. (d) Summary data showing the apoptosis rate in different groups. The cells at the early stage of apoptosis were stained with Annexin V+/PI−, and the cells at the late stage of apoptosis were stained with Annexin V+/PI+. The data are expressed as mean ± SD from three independent experiments. (^##^ *P* < 0.01, ^###^ *P* < 0.001 versus control, ^$^ *P* < 0.05; ^∗∗^ *P* < 0.01 versus DMSO).

After 24 h of culture, the postthawed NPMSCs cryopreserved with DMSO spread not well and some cells even exhibited shrunk morphology. Moreover, TUNEL staining revealed that the TUNEL-positive cells increased when cryopreserved with DMSO. As expected, ICA attenuated the morphological changes indicative of apoptosis (Figures 3(a) and 3(b)). To measure the apoptosis rate in postthawed NPMSCs, an Annexin V-FITC/PI apoptosis detection kit was used. The results suggested that the apoptosis rate of postthawed NPMSCs cryopreserved with DMSO was significantly higher than that of control (Figures 3(c) and 3(d), *P* < 0.001), and ICA could partially decrease the apoptosis rate. Interestingly, ICA mainly reduced the percentage of apoptotic cells at the early stage (Figures 3(c) and 3(d), *P* < 0.05).

### 3.3. Effect of ICA on Distribution of F-Actin and Cell Adherence in Postthawed NPMSCs

**Figure 4:**
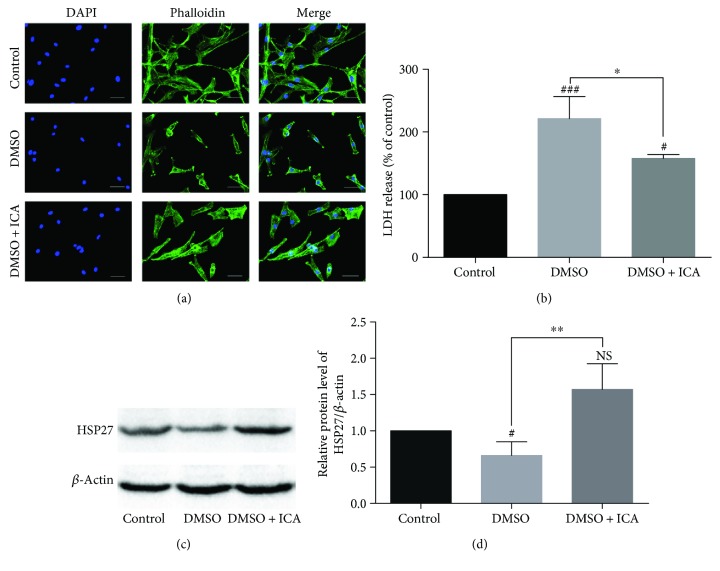
Analysis of cytoskeleton distribution and cellular membrane integrity of postthawed NPMSCs. (a) Representative fluorescence images of the distribution of cytoskeleton F-actin in different groups (bar = 50 *μ*m). (b) The release of LDH in different groups. (c) The typical western blot bands of HSP27. (d) Summary data showing protein levels of HSP27. The data are expressed as mean ± SD from three independent experiments. (^#^ *P* < 0.05, ^###^ *P* < 0.001 versus control; ^∗^ *P* < 0.05, ^∗∗^ *P* < 0.01 versus DMSO).

The fluorescence images showed that the noncryopreserved NPMSCs exhibited normal fibroblast cell-like shape and intact organization of F-actin after 24 h of culture. However, the loss of F-actin distribution and impaired cell adherence were observed in the postthawed NPMSCs cryopreserved with DMSO. And ICA restored the partial loss of F-actin distribution and improved the cell adherence (Figure 4(a)). The release of LDH was measured by a LDH-cytotoxicity assay kit, and the expression of HSP27 was analyzed by western blotting. The results indicated that LDH level was significantly higher and HSP27 expression was significantly lower in the postthawed NPMSCs cryopreserved with DMSO, supporting the presence of cellular membrane damages. However, ICA partially reversed the changes and maintained cellular membrane integrity (Figures 4(b)–4(d), *P* < 0.05).

### 3.4. ICA Inhibits the Decrease of MMP (ΔΨm)

**Figure 5:**
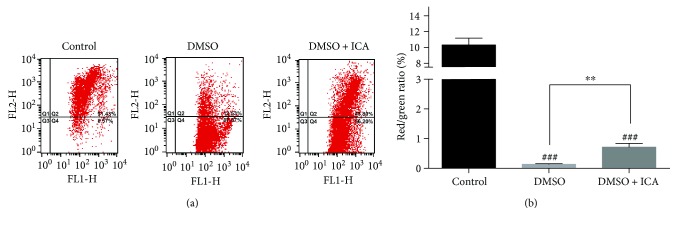
The addition of ICA maintained the ΔΨm of postthawed NPMSCs. (a) The MMP was analyzed by flow cytometry through JC-1 staining. (b) Summary data showing the quantitative MMP expressed as the ratio of red/green fluorescence intensity. The data are expressed as mean ± SD from three independent experiments. (^###^ *P* < 0.001 versus control, ^∗∗^ *P* < 0.01 versus DMSO).

Mitochondrial function of NPMSCs was evaluated by MMP assay. Compared to the noncryopreserved NPMSCs, the postthawed NPMSCs cryopreserved with DMSO exhibited a remarkable decrease of red/green fluorescence ratio, which indicated the reduction of ΔΨm and the presence of the damaged mitochondrial function. Not surprisingly, ICA significantly inhibited the decrease of ΔΨm and improved the mitochondrial function of postthawed NPMSCs (Figure 5, *P* < 0.01).

### 3.5. ICA Reduces Oxidative Stress of Postthawed NPMSCs

**Figure 6:**
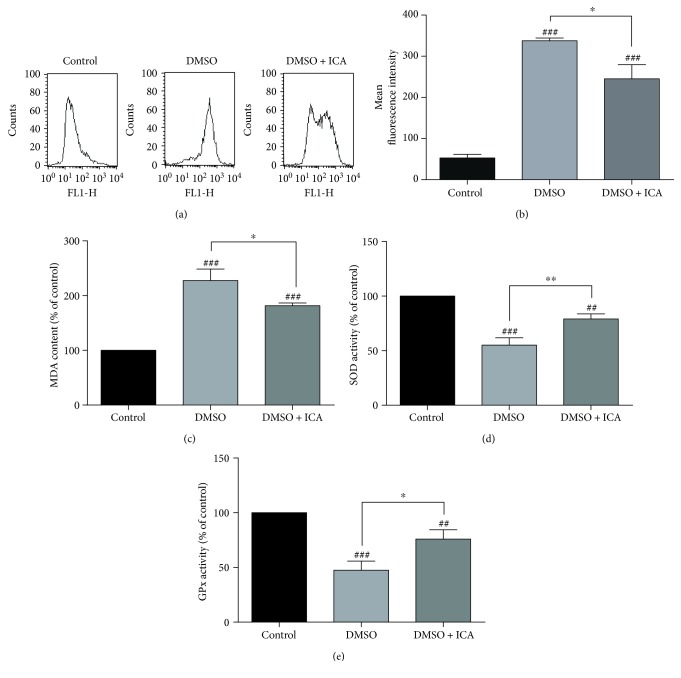
ICA inhibited the oxidative stress of postthawed NPMSCs. (a) Intracellular ROS levels were measured by flow cytometry through DCFH-DA staining. (b) Summary data showing the mean fluorescence intensity in different groups. (c) Intracellular MDA content in different groups. (d) SOD activity in different groups. (e) GPx activity in different groups. The data are expressed as mean ± SD from three independent experiments. (^##^ *P* < 0.01, ^###^ *P* < 0.001 versus control; ^∗^ *P* < 0.05, ^∗∗^ *P* < 0.01 versus DMSO).

To investigate whether the antioxidative function involved in the cell protective effect of ICA on the cryopreserved NPMSCs, the cellular contents of ROS, oxidation product MDA, and the reductive substances (SOD and GPx) were detected. As shown in Figure 6, a significant increase in ROS and MDA contents and a remarkable reduction in SOD and GPx activities were observed in postthawed NPMSCs cryopreserved with DMSO. However, ICA reduced the contents of ROS and MDA and improved the activities of SOD and GPx in postthawed NPMSCs (*P* < 0.05).

### 3.6. Effect of ICA on the Expression of Heat Shock Proteins in Postthawed NPMSCs

**Figure 7:**
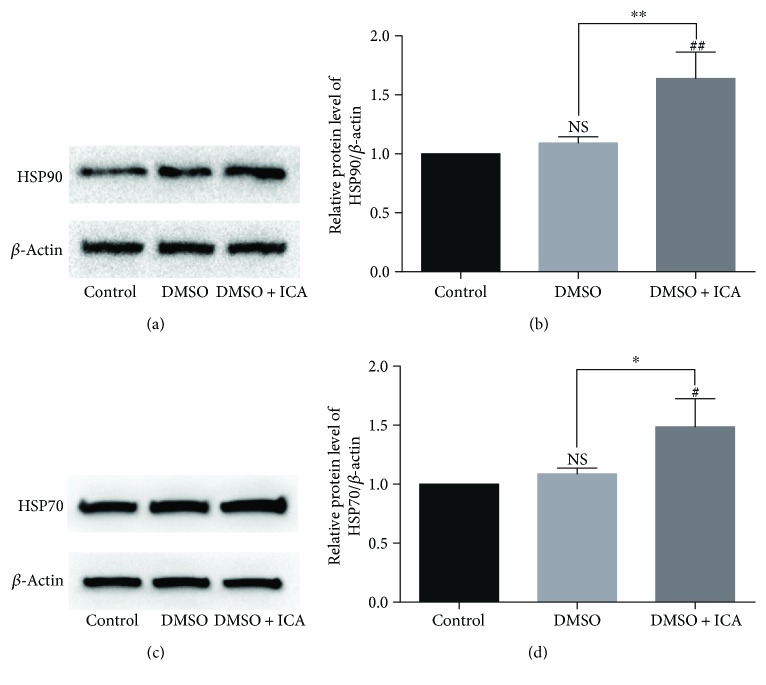
The protein expression of HSP90 and HSP70 determined by western blotting. (a, c) The typical western blot bands of HSP90 and HSP70. (b, d) Summary data showing protein levels of HSP90 and HSP70. The data are expressed as mean ± SD from three independent experiments. (^#^ *P* < 0.05, ^##^ *P* < 0.01 versus control; ^∗^ *P* < 0.05, ^∗∗^ *P* < 0.01 versus DMSO).

The expressions of HSP90 and HSP70 were analyzed by western blotting. The data suggested that ICA remarkably upregulated the expression of HSP90 and HSP70 of postthawed NPMSCs (Figure 7, *P* < 0.05). And there was no significant difference between the noncryopreserved NPMSCs in the control group with the postthawed NPMSCs cryopreserved with DMSO. The results demonstrated that higher expression of HSP90 and HSP70 in the presence of ICA might improve the viability and functionality of postthawed NPMSCs.

### 3.7. ICA Does Not Alter Characteristics of Postthawed NPMSCs

**Figure 8:**
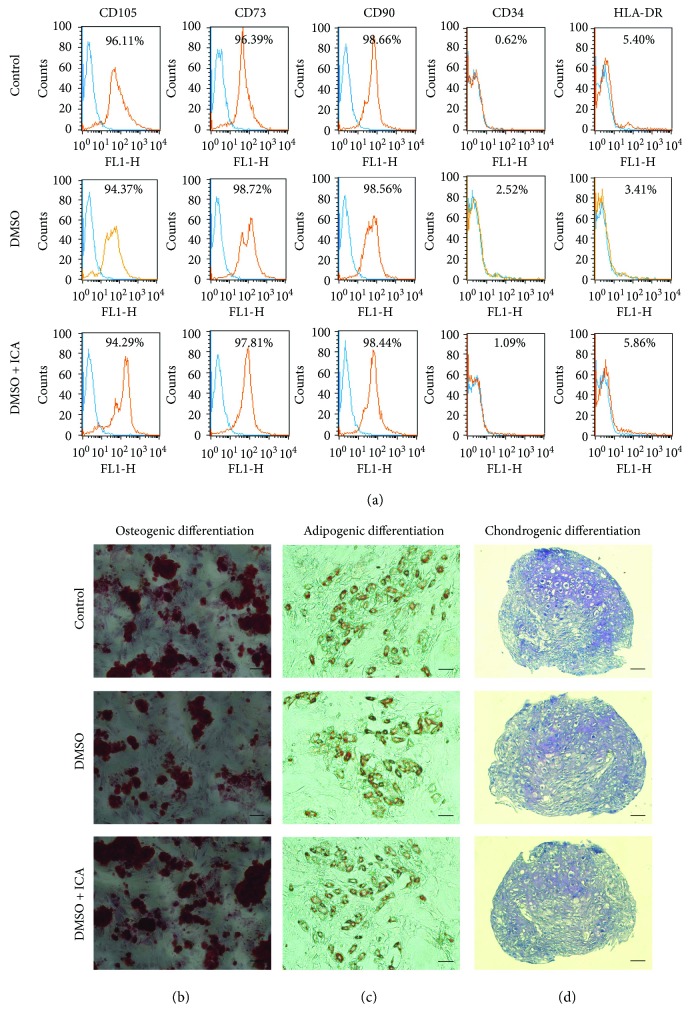
ICA had no influence on the characteristics of postthawed NPMSCs cryopreserved with DMSO and ICA. (a) The MSC-associated surface markers (CD105, CD73, CD90, CD34, and HLA-DR) were analyzed by flow cytometry. (b) Histological appearances with Alizarin Red staining (bar = 100 *μ*m). (c) Histological appearances with Oil Red O staining (bar = 100 *μ*m). (d) Histological appearances with Toluidine Blue staining (bar = 50 *μ*m).

Although cell protective effect of ICA on the cryopreserved NPMSCs was confirmed, whether ICA altered the characteristics of postthawed NPMSCs was unclear. To investigate whether ICA had influence on the characteristics of postthawed NPMSCs, we evaluated the expression level of MSC-associated surface markers and the multilineage differentiation potential of NPMSCs cryopreserved with DMSO and ICA. As shown in Figure 8, the NPMSCs cryopreserved with DMSO and ICA exhibited similar stem cell characteristics to those of noncryopreserved NPMSCs and cryopreserved NPMSCs with DMSO. More specifically, the NPMSCs cryopreserved with DMSO and ICA had high expression levels of markers (CD105, CD73, and CD90) that are positive in MSCs and had low expression of markers (CD34 and HLA-DR) that are usually negative in MSCs (Figure 8(a)). Meanwhile, the NPMSCs could be induced to differentiate into osteocytes, adipocytes, and chondrocytes (Figures 8(b)–8(d)).

## 4. Discussion

Recently, increasing evidences have demonstrated the existence of endogenous MSCs within IVD, including NPMSCs, annulus fibrosus-derived MSCs, and cartilage endplate-derived MSCs [31, 32]. Among them, NPMSCs play a major role in maintaining homeostasis and regeneration of degenerative IVDs [33]. More importantly, it was reported that endogenous NPMSCs showed great advantage in adaption to IVD-like adverse microenvironment in some vitro studies [34, 35]. Therefore, the application of endogenous NPMSCs provides a novel and promising strategy for the repair of IVD degeneration. However, freshly harvested and viable NPMSCs are not always available for basic research and clinical applications, and continuous supply and transportation of NPMSCs must rely on an effective cell cryopreservation technology.

In the conventional MSC cryopreservation process, cells are frozen in cryopreservation agents containing DMSO [36]. The MSCs are cooled at −1°C/min down to −80°C and then transferred into the liquid nitrogen tank. And the MSCs can be used after thawed in complete medium at 37°C [37]. Although this conventional cryopreservation method increases the access and availability of MSCs, there remain great challenges which need to be overcome. It was reported that the standardized cryoprotective agent DMSO could inhibit cell viability in vitro [38] and produce many toxic reactions in patients who received cryopreserved stem cell transplantation [39, 40]. And during cryopreservation, the changes in temperature and osmolarity would lead to the increase of ROS, mitochondrial injuries, cellular structural damages, and cell apoptosis [11]. Due to these cellular damages experienced during cryopreservation and thawing, the functionality of the MSCs would be affected negatively [41]. Thus, the optimal cryopreservation methods and better cryoprotective agents need to be developed to maintain the viability and function of postthawed MSCs.

In the present study, a novel formulation of cryopreservation media containing ICA along with DMSO was developed to explore the cell protective effect of ICA on cryopreserved human NPMSCs. The results showed that ICA had no cytotoxicity and could remarkably increase the viability of postthawed NPMSCs. And compared to cryopreservation media containing DMSO alone, the addition of ICA significantly decreased the apoptotic cell death in postthawed NPMSCs. In addition, it was reported that diminished adherence potential was related to the disruption of cytoskeleton integrity and the cellular membrane integrity, which could be indicated by the F-actin distribution and the release of LDH, respectively [16, 42]. The results in the study demonstrated that ICA partially reversed the impaired cell adherence of postthawed NPMSCs by improving the loss of F-actin distribution and decreasing the release of LDH. Furthermore, the data demonstrated that the addition of ICA significantly increased the ΔΨm and improved the mitochondrial function of postthawed NPMSCs. Taken together, the above results indicated that the addition of ICA to the conventional cryopreservation medium improved postthawed recovery of human NPMSCs.

Oxidative stress has been confirmed to exist in the freeze-thawing cells [41, 43]. During freeze-thawing process, excess ROS are accumulated. The excessive ROS production could result in an imbalance of the oxidation-reduction systems and lead to oxidative stress [43]. ICA could act as a ROS scavenger and exert antioxidative function to protect cells from oxidative stress [44]. Therefore, we assumed that ICA might improve the viability and function of cryopreserved human NPMSCs by inhibiting oxidative stress. To verify our hypothesis, we detected the intracellular contents of ROS and oxidation-reduction substances. The data showed that the addition of ICA significantly reduced the ROS production of postthawed NPMSCs. Meanwhile, MDA, a product of lipid peroxidation, decreased in the NPMSCs cryopreserved with ICA and DMSO. The data also showed that the addition of ICA remarkably increased the activity of SOD and GPx, which demonstrated the oxidation resistance [45]. Our results suggested that ICA could improve postthawed recovery of human NPMSCs, at least in part, by reducing oxidative stress.

HSPs are a family of proteins which function to facilitate protein folding and maintain the structures and functions of other proteins when cells are exposed to different kinds of stress conditions [46]. HSPs are classified based on the molecular weight and include large HSPs, HSP90, HSP70, HSP60, and HSP40 and small HSPs [47]. The HSP27, HSP70, and HSP90 classes of HSPs are widely studied in cryopreservation. It was reported that HSP27 expression was associated with the maintenance of cellular membrane integrity [42]. Our results suggested that ICA upregulated the expression of HSP27 and maintained the cellular membrane integrity of postthawed NPMSCs. The data in this study also suggested that addition of ICA could increase the expressions of HSP90 and HSP70, which have been suggested to correlate positively with postthawed MSC viability and functionality and can enhance the postthawed cell recovery of cryopreserved MSCs [16, 48]. These results indicated that the cell protective effect of ICA on the cryopreserved NPMSCs was related to the upregulation of HSPs.

Successful cryopreservation media can not only ensure the cell viability and function of postthawed cells but also preserve their characteristics [14]. Therefore, we evaluated the characteristics of postthawed NPMSCs cryopreserved with ICA and DMSO to investigate whether ICA altered the characteristics of postthawed NPMSCs. The results showed that the surface markers and multilineage differentiation potential of postthawed NPMSCs cryopreserved with ICA and DMSO were similar to those of noncryopreserved NPMSCs and fulfilled the criteria of MSC stated by the International Society for Cellular Therapy (ISCT) [49]. It proved that the addition of ICA had no influence on the characteristics of postthawed NPMSCs.

Certainly, there are a number of limitations to this study. First, the NPMSCs used in this study were obtained from degenerative NP tissues. It was reported that cells derived from degenerative and nondegenerative disc exhibited different biological behavior [50]. So, further studies with cells obtained from nondegenerative NP tissues need to be carried out. Second, the NPMSCs were cryopreserved for only one week in this research, and the cryopreservation time was short for clinical practice. Therefore, further studies with longer and different cryopreservation time are needed. Finally, we cryopreserved NPMSCs with ICA at a concentration of 25 *μ*M. But when we elevated the concentration of ICA to 50 *μ*M, the cell protective effect of ICA dropped. Some previous studies suggested that appropriate levels of ROS play important roles in normal biological processes [21, 51]. The reduced protective effect might be related to the excessive ROS-scavenging function of ICA at high concentration. So, the specific molecular mechanism remains to be explored.

In summary, the results in our study showed that ICA had multiple effects on postthawed NPMSCs, including increasing cell viability, decreasing apoptotic cell death, improving cell adherence, and maintaining the mitochondrial functions. And ICA might exert these protective effects by inhibiting oxidative stress and increasing the expression of HSPs. Furthermore, ICA had no cytotoxicity and could preserve the characteristics of postthawed NPMSCs. Therefore, adding ICA to conventional cryopreservation media may help to improve the viability and function of cryopreserved human NPMSCs.

## Data Availability

The data used to support the findings of this study are available from the corresponding author upon request.

## Supplementary Materials

Supplementary Materials Table S1: the characteristic details of the patients enrolled in the study. Table S2: the details about the use of samples enrolled in the study.
