# Supplementary material for: Icariin Improves the Viability and Function of Cryopreserved Human Nucleus Pulposus-Derived Mesenchymal Stem Cells
Source: Oxid Med Cell Longev. 2018 Jun 27;2018:3459612. doi: 10.1155/2018/3459612 (PMC6040248; doi:10.1155/2018/3459612)
Supplement: Supplementary Materials — Table S1: the characteristic details of the patients enrolled in the study. Table S2: the details about the use of samples enrolled in the study. [file 3459612.f1.pdf]

Table.S1

Characteristics details of the patients enrolled in the study.

Case no.	Age (years)	Gender	Diagnosis	Disc level	Pfirrmann grading
Case 1	43	F	Lumbar disc herniation	L5/S1	IV
Case 2	54	M	Lumbar disc herniation	L4/5	IV
Case 3	30	F	Lumbar disc herniation	L5/S1	III
Case 4	39	F	Lumbar disc herniation	L4/5	IV
Case 5	48	M	Lumbar disc herniation	L5/S1	IV

Table.S2

The details about the use of samples enrolled in the study.

Case no.	The separate components of the study
Case 1	Figure 2A-2B, 3A-3D, 6A-6E, 7A-7D, 8A-8B
Case 2	Figure 2A-B, 3A-3D, 4A-4D, 5A-5B, 6A-6E
Case 3	Figure 2A-2B, 3A-3D, 4A-4D, 5A-5B, 6A-6E
Case 4	Figure 4A-4D, 5A-5B, 6A-6E, 7A-7D, 8A-8B
Case 5	Figure 3A-3B, 4C-4D, 7A-7D, 8A-8B
